# Sclerotherapy as an esthetic indication in oral vascular malformations: a case series^☆^^☆☆^

**DOI:** 10.1016/j.abd.2019.09.010

**Published:** 2019-09-30

**Authors:** Brena Rodrigues Manzano, Aloizio Maciel Premoli, Natalia Garcia Santaella, Carla Renata Sanomiya Ikuta, Cássia Maria Fisher Rubira, Paulo Sérgio da Silva Santos

**Affiliations:** Department of Surgery, Stomatology, Pathology, and Radiology, Faculdade de Odontologia de Bauru, Universidade de São Paulo, Bauru, SP, Brazil

**Keywords:** Mouth, Sclerotherapy, Vascular malformations

## Abstract

**Background:**

The use of monoethanolamine oleate 5% is effective for the treatment of vascular malformations with low blood flow.

**Objectives:**

To report a case series of vascular malformations in the mouth and oral cavity treated with monoethanolamine oleate 5%.

**Methods:**

A retrospective descriptive study was performed in electronic patient charts covering seven years. Patient demographics, diagnostic resources, lesion site, size, and number of applications of monoethanolamine oleate 5% were collected.

**Results:**

A total of 21 vascular malformations were recorded, located mostly on the lower lip (52.3%) and resolved in a single application in 14 patients. The authors found 19 patients treated with sclerotherapy. Thirteen were women and six were men, with a mean age of 61 years.

**Study limitation:**

Small sample size.

**Conclusions:**

Sclerotherapy is an effective treatment for vascular malformations of the lips and oral cavity, with resolution after only one or two applications (*n* = 16).

## Introduction

Vascular malformations (VMs) are lesions of the vascular or lymphatic system that can affect any part of the body, but which are common on the head and neck region.^1^ Due to discrepancies in the literature as to the correct nomenclature, in 1996 the International Society for the Study of Vascular Anomalies (ISSVA) classified vascular anomalies as vascular tumors (hemangiomas) and vascular malformations, based on the lesions’ biological and pathological characteristics.^2^, ^3^

Vascular tumors develop through the vessels’ cell growth and proliferation. Meanwhile, VMs are characterized as a defect in the vessels’ maturation and vascular morphogenesis, caused mainly by a dysfunction in the regulation of the pathways in embryogenesis and vasculogenesis.^3^ VMs can be divided into low-flow and high-flow: low-flow VMs consist of a venous, capillary, or lymphatic component, while high-flow VMs consist of an arterial or arteriovenous component.^4^

VMs usually occur in children and young adults. In the oral cavity, they are normally observed on the lips, tongue, buccal mucosa, and palate.^5^, ^6^, ^7^ VMs do not regress spontaneously and can expand, and can be either single or multiple.^7^ The clinical manifestations consist of pain, ulcerations, bleeding, functional limitations, and esthetic alterations.^5^, ^8^, ^9^ The etiology may involve trauma or pregnancy and other hormonal factors that can induce the lesions’ growth.^3^

Treatment indication in VMs depends on a diagnosis that specifies the vascular type (arterial, venous, and/or lymphatic) and type of blood flow. The treatment options are: laser therapy, surgical excision, embolization, electrosurgery, and sclerotherapy combined with surgery.^7^, ^10^, ^11^

Sclerotherapy is a conservative technique that consists of intralesional injection of sclerosing agents, causing inflammation in the vessels followed by occlusion and vascular sclerosis, resulting in regression of the lesion.^12^ The sclerosing agents used to treat VMs include monoethanolamine oleate 5%, reported in the literature as a safe and efficient method with low toxicity for the treatment of these lesions in various regions of the body.^1^, ^13^, ^14^

The study aims to report 19 clinical cases of oral VMs that were treated with sclerotherapy using the application of monoethanolamine oleate 5% as monotherapy in VMs of the mouth, describing the diagnostic approach and therapeutic indication.

## Methods

### Characterization and data collection

This is a retrospective descriptive study based on collection of data from patients with oral VMs submitted to sclerotherapy with monoethanolamine oleate 5%. A search was conducted in the electronic patient file system, using the terms “vascular,” “vascular malformations,” and “vascular lesions,” from 2011 to 2017, resulting in 19 cases that were included in the current report. Demographic data were then collected, such as age, sex, and race of patients submitted to sclerotherapy. Data on the diagnostic resources used, location and size of the VMs, and number of applications of monoethanolamine oleate were also collected.

### Diagnostic criteria and sclerotherapy

Diagnosis of VM began with the clinical observation of painless purplish vesicles or bullae with a soft consistency on palpation. There were frequent reports of a pulsatile sensation and increased volume during or after physical exercise. History of the lesions featured reports of trauma, puberty, pregnancy, or hormonal alterations prior to the lesion. In all the cases, diascopy showed changes in coloring, intralesional ischemia, and decrease or alteration in the shape, corroborating the presumptive diagnosis of VM.

When the VM was 5 mm in diameter or smaller and well demarcated on visual inspection and palpation, the treatment of choice was sclerotherapy, without complementary diagnostic imaging tests. However, for lesions greater than 5 mm with imprecise limits on visual inspection and palpation or with multiple lesions in the same elective region for sclerotherapy, and in which the history of the current disease was inconsistent with VM and/or with clinical aspects suggestive of tumors, Doppler ultrasound was performed, which provided an adequate diagnosis according to type of vascular content and blood flow velocity.^4^ For cases in which the content was predominantly venous and low-flow, sclerotherapy remained indicated.

Application of the sclerosing solution followed the manufacturer's instructions. The sclerosing oil was diluted in anesthetic liquid at a proportion of 7:3 mL of monoethanolamine oleate 5% (0.7 mL) to mepivacaine 2% with epinephrine 1:100,000 (0.3 mL) in a 1 mL syringe. The first injection was applied in the central region of the VM, with introduction of the needle to a depth that included half the volume of the VM. Before injection of the sclerosing solution, positive aspiration of the blood vessel was verified to ensure correct intravascular application.

Ischemia and progressive increase in the pressure needed for the injection caused by the accumulation of the intralesional sclerosing solution were used as the criteria for interrupting application of the solution in that region. The needle/syringe was then redirected to another region of the VM where a new positive aspiration was observed before performing another injection. Per session, a maximum of 1 mL of sclerosing solution was applied, properly diluted, and for each application in a single region of the VM, the amount varied from 0.2 to 0.4 mL. Thus, per session, the sclerosing solution was injected in up to four different regions of a VM.

## Results

**Figure 1:**
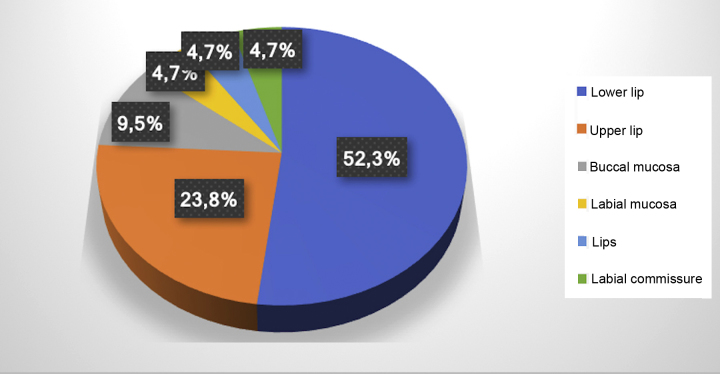
Distribution of location of the 21 oral vascular malformations.

The electronic patient chart search identified 19 patients that had undergone sclerotherapy in oral VMs, totaling 21 VMs, since two patients had two lesions. Mean age was 61 years, 13 patients (68%) were females, and 15 (79%) were white (Table 1). Lesions greater than 5 mm in diameter were found in six patients, and in these cases Doppler ultrasound was performed. In all the cases in this study, the VMs were of the venous type, with low blood flow. The most frequently affected region was the lower lip (52.3%), followed by the upper lip (23.8%), buccal mucosa (9.7%), labial mucosa (4.7%), both lips (4.7%), and labial commissure (4.7%) (Fig. 1).

**Table 1:** Distribution of patients treated with monoethanolamine oleate according to epidemiology and clinical characteristics of the lesions.

Patient	Age	Sex	Race	Symptomatology	Evolution	Associated factor	Location of VM
1	58	M	Black	Asymptomatic	27 months	Bit an object	Lower lip
2	63	F	White	Asymptomatic	10 months	Not reported	Buccal mucosa
3	62	F	White	Asymptomatic	10 years	History of varices	Upper lip
4	60	F	White	Asymptomatic	2 months	History of varices	Labial mucosa
5	49	M	White	Asymptomatic	20 years	Not reported	Lower lip
6	74	F	White	Asymptomatic	15 years	Not reported	Lower lip
7	62	M	White	Symptomatic	20 years	History of trauma	Upper lip
8	82	M	White	Asymptomatic	4 years	HRT	Upper lip
9	65	F	Not reported	Asymptomatic	10 years	HRT	Lips
10	61	F	Black	Asymptomatic	3 months	HRT	Lower lip
11	61	F	White	Asymptomatic	25 years	Bit her own lip	Lower lip
12	21	F	White	Asymptomatic	3 months	Not reported	Upper lip
13	48	F	White	Symptomatic	8 years	Not reported	Buccal mucosa
14	59	F	White	Not reported	2 years	History of trauma	Lower lip
15	68	F	Brown	Asymptomatic	40 years	Associated with previous pregnancy	Lower lip
							Labial commissure
16	63	F	White	Asymptomatic	6 years	History of trauma + HRT	Lower lip
17	63	M	White	Asymptomatic	2 years	HRT	Lower lip
18	69	F	White	Asymptomatic	1 year	Treatment for vascular disease	Lower lip
							Upper lip
19	64	M	White	Asymptomatic	4 years^a^	Not reported	Lower lip

HRT, hormone replacement therapy; VM, vascular malformation.

a As described on patient chart.

The number of sessions varied according to the decrease in coloring and volume of the VMs, and especially with each individual patient's esthetic satisfaction. The patients returned for follow-up assessment, and when necessary a new session with application of the sclerosing solution was performed 20 days after the last injection. One to five sessions were performed in each lesion until obtaining a satisfactory result, and most required one (38.1%) or two (38.1%) applications of sclerosing solution. In three lesions (14.3%), three applications were performed; one lesion (4.75%) required four applications; and only one case (4.75%) required five applications (Table 1).

**Figure 2:**
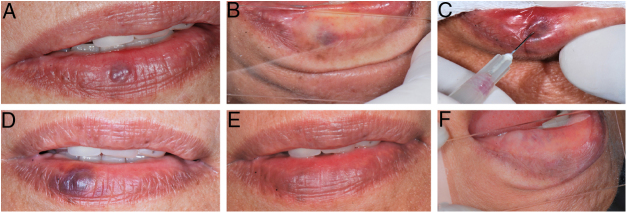
(A) Initial clinical appearance of vascular malformation, purplish vesicle measuring 3 mm on lower lip (patient 16); (B) positive diascopy for vascular lesion performed during clinical examination; (C) application of monoethanolamine oleate 5% with positive aspiration of blood in the vascular lumen; (D) clinical appearance of lesion on seventh day post-procedure; (E) final clinical appearance of vascular lesion on 28th day post-procedure; (F) final negative diascopy of the region where the VM was previously located.

During the procedure, all the patients complained of a local burning sensation, and edema and mild ischemia were observed in the region. In the first post-procedure consultation, all the patients reported a sensation of hardening in the lesion's area, but only during the first 72 h after the procedure; only one patient reported tingling, and one patient also noted paresthesia in the region, but there were no complaints in the subsequent follow-up visits. In all the cases, there was a reduction in the volume and fading of the VMs, with a report of esthetic improvement in the treated area (Fig. 2).

## Discussion

In the current study, the VMs were found in adults and elderly, differing from the findings in the literature showing that they were more frequent in young people and young adults.^15^, ^16^, ^17^ They were more prevalent in women, corroborating other reports.^7^, ^17^ As for location, the VMs were found more frequently on the lips, also in agreement with the case series in the reviewed literature.^5^, ^6^, ^16^

VMs can be classified according to blood flow velocity, which can be slow (venous, lymphatic, and capillary VMs) or rapid (arterial and arteriovenous VMs).^2^, ^18^ Venous VMs appear on Doppler ultrasound as hypoechoic tubular structures, demonstrating a slow blood flow pattern.^19^ Correct diagnosis is essential for the success of sclerotherapy, since efficient treatment requires prolonged contact between the sclerosing agent and the vascular wall; thus, sclerotherapy is most effective and safer in low-flow venous VMs.^15^

In this case series, VMs were diagnosed through a detailed clinical examination, with the semiotic procedure of diascopy, and by Doppler ultrasound in lesions greater than 5 mm. Thus, in all the cases the VMs were venous with slow blood flow, indicating sclerotherapy. In cases where the VM presented large caliber arteriovenous content with high blood flow, sclerotherapy was contraindicated.

Sclerotherapy is the treatment of choice for VMs, with a success rate of 70%–100%.^1^, ^16^, ^20^, ^21^, ^22^, ^23^, ^24^, ^25^ For smaller lesions, the treatment can lead to complete remission of the lesion or a significant reduction in the size, without the need for complementary therapies.^18^ Among the various sclerosis-inducing agents, monoethanolamine oleate is currently the best option due to the low toxicity in comparison to other sclerosing agents.^13^, ^14^ In addition, this type of treatment has lower odds of hemorrhage, scarring, and hyperpigmentation, besides being less invasive than surgery.^1^, ^25^

There is still no consensus in the literature as to the ideal concentration of this sclerosing agent for the treatment of VMs.^1^, ^7^, ^15^, ^16^ A previous study indicated the use of monoethanolamine oleate 5% at lower doses than 1 mL per session.^26^ The Brazilian National Health Surveillance Agency (Agência Nacional de Vigilância Sanitária [ANVISA]; 2016) recommends a dose of 0.5–2 mL in each vein, with the total not to exceed 6 mL. In this study, 0.7 mL of monoethanolamine oleate was used at a concentration of 5% diluted in 0.3 mL of anesthetic, totaling 1 mL maximum per session in each lesion, which is the protocol used at this institution.^27^

In 2005, Hyodoh et al. reported that a single application was sufficient for total resolution in smaller vascular lesions (≤30 mm). However, in the study, all the vascular lesions were smaller than 30 mm, but only eight cases (42%) received a single application of the sclerosing agent.^14^ On average, 1.6 sessions were needed for each lesion using monoethanolamine oleate at a concentration of 5% until resolution of the lesions, the same method already used in previous studies.^15^, ^16^, ^25^ The 5% concentration was responsible for the smaller number of sessions when compared to the study by Johann (2005), who required an average of 3.7 sessions due to the use of lower concentrations of the sclerosing agent (1.5% and 2.5%).^1^

Complications in sclerotherapy are dose-dependent, and the most common one is peripheral nerve damage or skin necrosis, which can result in scars and lead to esthetic problems.^15^, ^28^, ^29^, ^30^ This condition occurs when an excessive amount of the sclerosing agent invades the normal tissue or when high pressure is used during the application.^25^, ^30^

Patients may also develop hemoglobinuria and hemolytic renal failure, but this is limited to doses greater than 9.6 mL of monoethanolamine oleate.^31^, ^32^ Thus, in this study, the sclerosing solution was injected with light pressure in the vascular lumen, using positive aspiration of blood to be certain of depositing the agent in the correct place to avoid or minimize complications.

Only two patients presented symptoms in the area of the VMs at the time of the initial evaluation, and all of the patients had complained of unsatisfactory esthetic appearance before treatment. Therefore, sclerotherapy was an esthetic indication for the cases treated in this study. All patients reported a burning sensation during application of the sclerosing solution, which had also been described in other studies.^1^, ^15^ In addition, in 2002, Choi et al. reported burning sensation during injection even in cases in which local anesthetic was applied prior to sclerotherapy, but there were no complaints of pain during infiltration of the sclerosing agent in any of the cases.^15^ In 2007, Bonan observed that dilution of monoethanolamine oleate in distilled water also does not reduce the symptoms; besides, it could be responsible for the decrease in action by the chemical agent on the vessel walls.^33^

It was also observed and reported that after application of the sclerosing solution, there was edema that lasted from 72 h to seven days after the procedure. Other post-procedure symptoms were paresthesia in one patient (5.2%) and one case of tingling (5.2%). These signs and symptoms had resolved spontaneously by the first follow-up visit, with no need for additional therapy. No other side effects were observed in this study, and the complications rate did not differ from that of other studies.^1^, ^7^, ^15^, ^25^, ^34^

In all the VMs (21 lesions), the application of 0.7 mL monoethanolamine oleate 5% as monotherapy was an easy, simple, quick method, well tolerated by patients, with low morbidity, since it was performed as an outpatient procedure with a limited number of sessions. Despite the small sample size and the fact that this was a retrospective study with the analysis of electronic patient files, the data showed that this is an effective method in 100% of the cases, since it showed improvement in the clinical signs and symptoms, with a subjective and objective decrease in the volume or swelling of treated VMs and thus an esthetic improvement in the region.

## Conclusion

Sclerotherapy with monoethanolamine oleate 5% as monotherapy is effective, safe, and easy to apply, capable of providing excellent esthetic results within a small number of sessions, and is well accepted by patients.

## Financial support

This study was financed in part by the Coordenação de Aperfeiçoamento de Pessoal de Nível Superior - Brasil (CAPES) - Finance Code 001.

## Author's contribution

Brena Rodrigues Manzano: Conception and planning of the study; obtaining, analyzing, and interpreting the data; critical literature review; critical review of the manuscript.

Aloizio Maciel Premoli: Elaboration and drafting of the manuscript; effective participation in the research orientation; intellectual participation in the propaedeutic and/or therapeutic management of cases; critical review of the manuscript.

Natalia Garcia Santaella: Approval of the final version of the manuscript; elaboration and drafting of the manuscript; critical review of the manuscript.

Carla Renata Sanomiya Ikuta: Approval of the final version of the manuscript; elaboration and drafting of the manuscript; intellectual participation in the propaedeutic and/or therapeutic management of cases; critical review of the manuscript.

Cássia Maria Fisher Rubira: Approval of the final version of the manuscript; intellectual participation in the propaedeutic and/or therapeutic management of cases; critical review of the manuscript.

Paulo Sérgio da Silva Santos: Approval of the final version of the manuscript; conception and planning of the study; intellectual participation in the propaedeutic and/or therapeutic management of cases; critical review of the manuscript.

## Conflicts of interest

None declared.
